# Induced Pluripotent Stem Cells for Traumatic Spinal Cord Injury

**DOI:** 10.3389/fcell.2016.00152

**Published:** 2017-01-19

**Authors:** Mohamad Khazaei, Christopher S. Ahuja, Michael G. Fehlings

**Affiliations:** ^1^Division of Genetics and Development, Krembil Research InstituteToronto, ON, Canada; ^2^Institute of Medical Science, University of TorontoToronto, ON, Canada; ^3^Division of Neurosurgery, University of TorontoToronto, ON, Canada; ^4^Spinal Program, Toronto Western Hospital, University Health NetworkToronto, ON, Canada; ^5^Faculty of Medicine, University of TorontoToronto, ON, Canada

**Keywords:** spinal cord injury, stem cell, induced pluripotent stem cell, iPS, trauma, neural precursor cell

## Abstract

Spinal cord injury (SCI) is a common cause of mortality and neurological morbidity. Although progress had been made in the last decades in medical, surgical, and rehabilitation treatments for SCI, the outcomes of these approaches are not yet ideal. The use of cell transplantation as a therapeutic strategy for the treatment of SCI is very promising. Cell therapies for the treatment of SCI are limited by several translational road blocks, including ethical concerns in relation to cell sources. The use of iPSCs is particularly attractive, given that they provide an autologous cell source and avoid the ethical and moral considerations of other stem cell sources. In addition, different cell types, that are applicable to SCI, can be created from iPSCs. Common cell sources used for reprogramming are skin fibroblasts, keratinocytes, melanocytes, CD34+ cells, cord blood cells and adipose stem cells. Different cell types have different genetic and epigenetic considerations that affect their reprogramming efficiencies. Furthermore, in SCI the iPSCs can be differentiated to neural precursor cells, neural crest cells, neurons, oligodendrocytes, astrocytes, and even mesenchymal stromal cells. These can produce functional recovery by replacing lost cells and/or modulating the lesion microenvironment.

## Introduction

Traumatic SCIs result in devastating disability for over one million people in North America alone. With direct lifetime costs exceeding $1.1–4.6 million USD per patient, the importance of developing an effective regenerative treatment for SCI cannot be overstated (National Spinal Cord Injury Statistical Center, 2014). Cell-based therapies are an exciting strategy to address this pressing need. Unfortunately, there is no readily accessible source of autologous primary CNS cells, and embryonic stem cells (ESCs) have a diminishing role given limited supplies and ethical concerns. As a result, induced pluripotent stem cells (iPSCs) have emerged as a promising approach given their potential to generate autologous iPSCs and the virtually limitless supply available for research and treatment (Ahuja and Fehlings, 2016). This article provides a primer on the challenging pathophysiology of SCI and highlights key iPSC-based techniques with the greatest potential for translation over the coming decade.

## Pathophysiology

SCI has a unique pathophysiology characterized by an initial traumatic insult (primary injury) followed by a rapid and progressive secondary injury cascade which generates further permanent damage (Choo et al., 2007; LaPlaca et al., 2007). Early cell death occurs due to cell permeabilization, ischemia, and an overwhelming increase in pro-apoptotic signaling. This is compounded by disruption of the sensitive blood-spinal cord barrier leading to a marked influx of peripheral inflammatory cells, pro-inflammatory cytokines, and fluid shifts causing spinal cord swelling (Mautes et al., 2000; Whetstone et al., 2003). Over the following hours, by-products of cell necrosis (DNA, ATP, glutamate) are released into the microenvironment leading to further cell death and activation of pro-inflammatory microglia. Together, this leads to *en masse* infiltration of macrophages and additional microglia which generate cytotoxic reactive oxygen species as they phagocytose debris. Neutrophils and later lymphocytes also infiltrate the normally immune-privileged cord parenchyma and cyclically add to the inflammatory response (Waxman, 1989; Ulndreaj et al., 2016).

**Figure 1:**
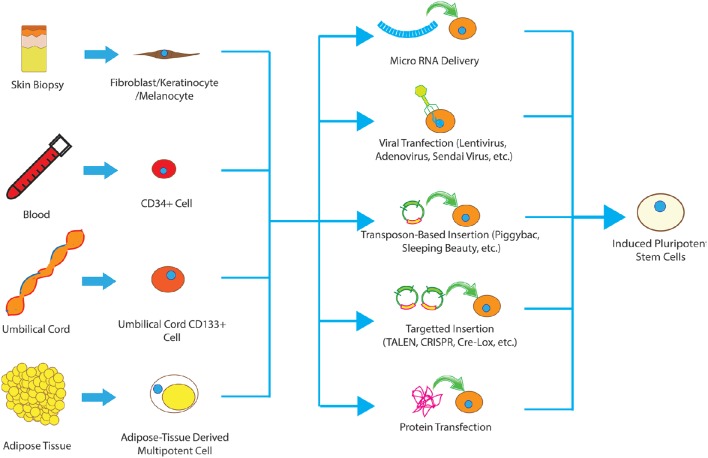
**Different types of cells have been used to produce iPSCs, including fibroblasts, keratinocytes, melanocytes, CD34+ cells, cord blood cells, and adipose stem cells**. These somatic cells can be reprogrammed to pluripotent state using viral methods, microRNA, transfection of reprograming proteins, episcopal vectors and integrating vectors. The collective term for the resultant cells is induced pluripotent stem cells.

Over the subsequent weeks to months, inflammation begins to subside leaving a severely disrupted neural and structural architecture. Loss of oligodendrocytes results in segments of demyelinated and dysfunctional tracts which begin to die back from the site of injury. Neurons attempt to regenerate but are impeded by an interwoven network of hyperproliferative astrocytes, known as the glial scar, which surround the lesion epicenter. The normal extracellular matrix now also contains dense deposits of chondroitin sulfate proteoglycan (CSPGs) which form a formidable barrier to neurite outgrowth. Furthermore, the loss of tissue volume leads to the formation of microcystic cavitation which coalesces into large regions devoid of an extracellular substrate for migration and growth. While the lesion continues to develop over years, attempts at regeneration by endogenous cells are severely hindered by these barriers (Figure 1; Ahuja et al., 2016).

These harsh post-injury conditions have been a challenge for cell-based regenerative therapies making optimization of the transplanted cells critical to success.

## Cell therapy for SCI

Numerous pluripotent and multipotent cell types have been investigated in SCI. The therapeutic potential of each varies depending on their cellular behavior, post-transplantation survival and proliferation, and unique differentiation profile. The purported mechanism of action for each cell type also differs but generally they fall into broad categories of regeneration of lost neurons, remyelination of axons, trophic support, immune modulation, modification of the extracellular environment, or a combination thereof (Tobias et al., 2003; Tetzlaff et al., 2011; Vawda et al., 2012). Importantly, the partially or fully differentiated progeny of iPSCs act through all of these mechanisms depending on the cell type, highlighting the broad utility of the technology.

## Induced pluripotent stem cells

Isolation and expansion of multipotent and differentiated autologous cells is difficult and time consuming. Furthermore, there is no readily accessible source of autologous CNS cells. For this reason, many cell-based therapies have utilized ESCs, however, limited supplies and ethical concerns have been a significant challenge with this option. Induced pluripotent stem cells (iPSCs) were generated by Yamanaka and colleagues in 2006 (Takahashi and Yamanaka, 2006). They showed that pluripotent stem cells, with properties similar to ESCs, could be generated from mouse fibroblasts by the simultaneous introduction of four factors (Oct4, Sox2, Klf2, and c-Myc; Takahashi and Yamanaka, 2006). In 2007 they reported that a similar approach could be used to generate human iPSCs from human fibroblasts (Takahashi et al., 2007). Concurrently, James Thomson's group reported on the generation of human iPSCs with an alternative combination of factors including Oct4, Sox2, Nanog, and Lin28 (Yu et al., 2007). Together, this work heralded a new age in stem cell research for SCI as supplies were limitless, adult-derived, and could potentially be made autologous.

## Generating iPSCs

Since the introduction of iPSCs, numerous protocols utilizing different combinations of transcription factors with varying efficiency rates have been published (Zhao et al., 2008; Meng et al., 2012). A key to translation is to ensure that the generation of iPSCs is robust, consistent, and safe. Several labs are currently optimizing iPSC protocols, including choice of transcription factor and route of delivery (e.g., viral, plasmid, etc.), to allow the cells to be used for clinical trials. This requires a balance between efficiency of generation and safety. For example, most preclinical protocols employ the proto-oncogene c-Myc, however, this would be highly concerning in clinical use given its prominent role in breast, ovarian, colorectal, pancreatic, and gastric cancers (Chen et al., 2014). As a result, other factors need to be used which thus far have resulted in lower reprogramming efficiency (Nakagawa et al., 2008).

The method used to introduce transcription factors into the cells is also important for clinical translation. Traditionally, lentivirus has been the vector of choice, however, integration of lentiviral DNA into actively replicating regions of the host genome presents safety concerns. Other viruses such as Sendai Virus and (Ban et al., 2011) adenovirus (Zhou and Freed, 2009) have also been used with potentially lower risks but much lower efficacy. As a result, several non-viral methods have been developed and validated for induction of pluripotency including Episomal vectors (Subramanyam et al., 2011), recombinant proteins (Yu et al., 2009), mRNAs (Kim et al., 2009), micro-RNAs (Warren et al., 2010), and removable transposons (e.g., piggyBac; Woltjen et al., 2009; Figure 1).

## Optimizing the source of iPSCs for SCI

Several somatic cell types have been used to successfully produce iPSCs including fibroblasts, keratinocytes, melanocytes, CD34+ cells, hepatocytes, umbilical cord blood cells, and adipocytes. While the resultant iPSCs all display the hallmarks of pluripotency, epigenetic modifications within the somatic cell of origin are retained even in the induced pluripotent state termed “epigenetic memory.” These modifications, including DNA methylation, histone acetylation, histone phosphorylation, and many others, result in a preference of iPSCs to follow the cell of origin in both gene expression and differentiation profile (Kim et al., 2011). For example, human keratinocyte-derived iPSCs have a higher tendency to differentiate to NPCs than less-invasively obtained CD34+ cells from blood, likely due to a common ectodermal germ layer origin (Kim et al., 2011).

Another important factor to consider is the reprogramming efficiency of each cell type. Maintained expression of key reprogramming factors enhances the frequency of successful reprogramming significantly. For example, keratinocytes can be reprogrammed to pluripotent cells at much higher frequency and a faster rate than fibroblasts from the same biopsy sample, likely due to greater existing Klf4 and c-Myc expression (Colman and Dreesen, 2009). This underscores the importance of selecting the appropriate starting cell type for SCI treatment balancing epigenetics, ease of harvesting, and the intended final product. Below we described the most commonly employed cells of origin and discuss in greater detail their advantages and disadvantages (Figure 1).

### Skin fibroblasts

Skin fibroblasts are one of the most commonly used cell sources for reprogramming. Adult human fibroblasts can be easily obtained, purified, and maintained in culture (Maherali et al., 2008; Chen et al., 2013a) which is ideal for autologous transplantation in patients with SCI. However, skin fibroblast reprogramming to iPSCs is lengthy due to low reprogramming efficiency. Three to four weeks are required for the amplification of the fibroblasts derived from a human skin biopsy (Park et al., 2008) and another 3–4 weeks for iPSC colonies to appear (Park et al., 2008). After 2 months in culture, only ~0.01% of adult human skin fibroblasts become iPSCs if the four Yamanaka factors are used and this number drops further if three or fewer factors are employed (Huangfu et al., 2008). Yamanaka suggested that fibroblasts, as terminally differentiated cells, required much more aggressive reprogramming than less differentiated cells (Yamanaka, 2009) increasing the cost (i.e., hands-on time and reagents) and putting the cells at greater risk of mutation due to a longer time in culture.

### Keratinocytes

Keratinocytes have emerged as a promising cell source for reprogramming because they can be easily accessed via a small skin biopsy or plucked hair (Aasen and Izpisúa Belmonte, 2010) making the generation of autologous iPSCs easier for future clinical trials. Keratinocytes do require more time than fibroblasts to expand but they can be reprogrammed much more quickly (10 days) and with greater efficiency (Aasen et al., 2008). The higher reprogramming efficiency of keratinocytes is mainly due to the higher basal levels of Klf4 and c-Myc (Aasen et al., 2008).

### Melanocytes

Similarly, melanocytes can also be isolated from skin biopsies. Melanocytes contain high levels of endogenous Sox2 and therefore only require the other three factors for reprogramming (Utikal et al., 2009). Furthermore, only 10 days in culture are needed for melanocytes to be reprogrammed and they have shown a reprogramming efficiency of 0.19% (Utikal et al., 2009). All of this suggests that melanocytes may be a viable option as a cell source for autologous iPSC transplants when they are needed very quickly. However, it remains unknown whether melanocyte-derived iPSCs can be differentiated to NPCs, what the differentiation potential of these NPCs would be and whether they may be used in SCI.

### Cord blood cells

Umbilical cord blood has been used to generate iPSCs because the source tissue is often discarded postpartum otherwise and there are many umbilical cord blood banks around the world containing potentially autologous (or 1st degree relative) cells. These cells can be cryopreserved for prolonged periods and have been shown to maintain their ability to generate iPSCs for many years (Giorgetti et al., 2009, 2). CD133+ cells from umbilical cord blood can be reprogrammed to iPSCs by using as few as two factors (Oct4 and SOX2) with a reprogramming efficiency of 0.45% (Giorgetti et al., 2009, 2). Cells isolated from umbilical cord blood are in a primitive state and are therefore ideal for reprogramming due to their epigenetic signature as they may be closer than other differentiated cells to the pluripotent state (Red-Horse et al., 2004). However, the disadvantage of iPSCs from the umbilical cord is that their preferred differentiation lineages may not match what is required for CNS regeneration as they originate in a different germ cell layer.

### CD34^+^ cells

Cells expressing CD34 are typically found in bone marrow and umbilical cord blood and have been used successfully to generate human iPSCs (Loh et al., 2009). Several populations of blood-borne CD34+ cells exist in small numbers including hematopoietic stem cells, B-cell precursors, and megakaryocytes (Sidney et al., 2014). Unfortunately, the number of these cells in circulation at any one time tends to be quite low, often necessitating the use of granulocyte-colony stimulating factor (G-CSF) stimulation of bone marrow (Loh et al., 2009). While well-studied and commonly used in patients after chemotherapy, G-CSF is associated with its own complications including rashes, fever, fatigue, splenomegaly, and allergic reactions (Brockmann et al., 2013) which may be significant for patients with SCI. The reprogramming of CD34+ cells is also inefficient at 0.01–0.02% with the Yamanaka Factors (Loh et al., 2009). This efficiency further drops for CD34+ cells isolated from umbilical cord blood (Ramos-Mejía et al., 2012) making the utility of CD34+ reprogramming for clinical use controversial.

### Adipose tissue derived stem cells

Fat-derived stem cells are abundantly available after liposuction procedures (Bunnell et al., 2008). As many as 100 million cells can be isolated from a 300 mL sample and can be expanded for reprogramming in ~48 h (Sun et al., 2009). Using Yamanaka Factors, adipose tissue stem cells can be reprogrammed in 10–15 days with an efficiency of 0.2% (Sun et al., 2009). Furthermore, these cells have high intrinsic levels of Klf4 and their multipotent nature may mean they require fewer epigenetic changes to reach pluripotency (Qu et al., 2012). This has made fat-derived stem cells an exciting area of research for the generation of iPSCs.

## Differentiated iPSCs for SCI

After generation of iPSCs, the cells must be differentiated to the appropriate multipotent or fully differentiated cell type for the treatment of SCI. If left in an undifferentiated state, there may exist a risk of teratoma formation due to the ability of these rapidly dividing cells to become all three germ layers *in situ*. In fact, incomplete differentiation and/or purification to remove undifferentiated cells is one of the biggest safety concerns with clinical translation of iPSC-derived therapies for SCI. Our lab and others have developed techniques to generate safe and effective monoclonal NPCs from iPSCs using non-viral methods. These cells have also been shown to survive in animal models of SCI without evidence of tumor formation or hyperproliferation (Tropepe et al., 2001; Smukler et al., 2006; Rowland et al., 2011; Chaddah et al., 2012).

**Figure 2:**
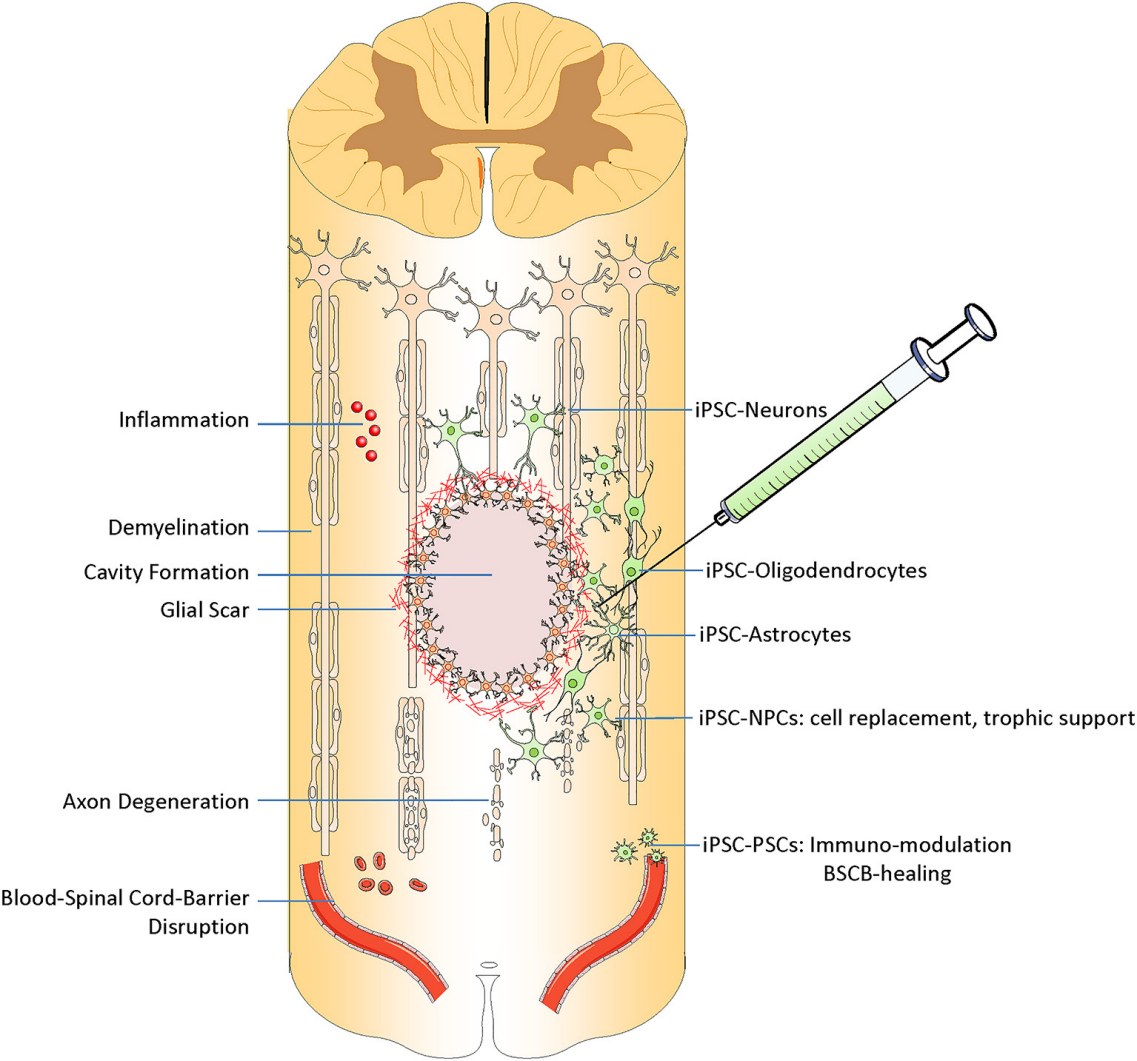
**Transplantation of iPSC derived cells can target different pathophysiological aspects of spinal cord injury**. After injury, spinal cord experiences inflammation, demyelination, and formation of cystic cavity, glial and fibrotic scaring, axonal degeneration and neural cell death, and disruption of blood spinal cord barrier BSCB. iPSC derived cells can replace the lost neurons, oligodendrocyte, and astrocytes. They can promote demyelination, modulate the immune response and also promote the BSCB healing.

Below we review the most clinically-relevant iPSC-derived cell types being used in preclinical research (Figure 2).

### iPSC-derived NPCs

NPCs are one of the most promising cell types that have been studied thus far in the treatment of SCI due to their ability to replace lost circuits as neurons, remyelinate axons as glia, and provide local trophic support (Tsuji et al., 2010; Nori et al., 2011; Kobayashi et al., 2012). Several protocols have been developed to differentiate iPSCs to NPCs effectively such as dual SMAD inhibition (Chambers et al., 2013) or embryoid body formation followed by differentiation into neural rosettes (Muratore et al., 2014). NPCs can also be generated directly from somatic cells without an intermediate pluripotent state. The Wernig lab is a pioneer in generating directly reprogrammed NPCs (drNPCs) by using Sox2, FoxG1, and Brn2 reprogramming factors (Lujan et al., 2012). Several other combinations of factors that can directly reprogram somatic cells to NPCs have subsequently been discovered (Han et al., 2012; Ring et al., 2012; Zou et al., 2014).

In our laboratory, we have recently been able to generate definitive NPCs utilizing non-viral piggyBac transposon-induced iPSCs and induction of the notch pathway. We have shown that this method is safe and effective (Salewski et al., 2013) and the resulting autologous iPSC-NPCs could be transplanted into a thoracic (T6-level) clip-contusion model of SCI in mice (Salewski et al., 2015). Interestingly, the transplanted cells integrated well with host tissue and differentiated primarily to remyelinating oligodendrocytes (59%). This resulted in significant functional recovery of locomotion as assessed by open field gait analyses (Salewski et al., 2015). Other studies using iPSC-NPCs in rodents and primate models of SCI have also found that transplanted cells can differentiate into neurons and glia *in vivo* resulting in enhanced remyelination, axon regeneration, tissue sparing, and behavioral outcomes (Fujimoto et al., 2012; Kobayashi et al., 2012; Nutt et al., 2013).

These effects are not limited to the site of transplant. A key study has shown that iPSC-NPCs transplanted into the spinal cord parenchyma of NOD-SCID mice can migrate significant distances (Fujimoto et al., 2012). The neurons derived from these cells were able to integrate into the host tissue and most commonly acted as interneurons forming synapses to reconstruct local neuronal circuits. This study validated hiPSCs-NPCs as a translationally-relevant neural cell source for preclinical research and was an important step toward clinical use.

In a 2016 study by Tuszynski lab, iPSC- and ESC-derived NPCs with a spinal cord specific identity were generated and transplanted. This study found that cells with a spinal cord identity effectively promoted corticospinal tract regeneration and contributed to functional recovery better than classically-derived NPCs which express markers of brain identity (Kadoya et al., 2016). This studies further highlights the importance of matching the identity of transplanted cells with the niche of the host tissue.

### iPSC derived neurons

Although transplantation of mature neurons into the spinal cord has typically resulted in poor integration and plasticity of transplanted cells as compared to neural progenitors, this is still a growing area of research requiring further optimization. iPSCs have been differentiated to several types of functional neurons including dopaminergic neurons (Zhang et al., 2013; Hallett et al., 2015), cerebral cortical neurons (Shi et al., 2012a,b), motor-neurons (MN)s (Sareen et al., 2013), and GABAergic interneurons (Nicholas et al., 2013). In SCI, there is major loss of motorneruons and interneruons and although transplantation of neurons with a cerebral cortical identity (Shi et al., 2012a,b) has beneficial effects, it is speculated that transplantation of neurons which possess a spinal cord regional identity can more effectively engraft into the spinal cord neural circuitry. In a recent study by Fandel et al. transplantation of stem cell-derived GABAergic interneurons (Fandel et al., 2016) into the lumbar cord at 2 weeks after thoracic SCI resulted in synaptic connections within the local circuitry and alleviated long-term neurogenic bladder dysfunction and neuropathic pain. Stem cell-derived motor neurons are increasingly being used for cellular replacement strategies in SCI. Motor neurons (MNs) and motor neurons precursor cells (MNPs) have successfully been generated from iPSCs and their use is particularly exciting as this key population of cells is required in small numbers to effect significant functional benefit (Sareen et al., 2013). Several protocols have been established for the generation of MNs from iPSCs. Some force the expression of MN specific factors (neurogenin 2, islet-1, and LIM/homeobox protein 3) in iPSCs derived from human fibroblasts while others rely on caudalization and centralization morphogens to generate MNPs (Jha et al., 2015). Other successful protocols have used a combination of reprogramming factors and growth factors such as GDNF, BDNF, and CNTF to produce MNs efficiently (Karumbayaram et al., 2009).

### iPSC derived astrocytes

After SCI, both gray and white matter astrocytes are lost over a distance of several millimeters. Although reactive astrocytes proliferate, form a glial scar, and secrete inhibitory agents such as CSPG, there have been reports that during the first weeks after injury reactive astrocytes actually protect tissue and contribute to some of the initial spontaneous recovery in patients. This likely stems from astrocytes' capacity to form a barrier around the lesion epicenter thereby containing infiltrating peripheral inflammatory cells through the compromised blood-spinal cord barrier and limiting the spread of the secondary injury cascade (Faulkner et al., 2004; Renault-Mihara et al., 2008). Furthermore, transplanted cells that differentiate to immature astrocytes may facilitate axon regeneration by providing trophic support and depositing structural extracellular matrix proteins (Tsuji et al., 2010). In fact, transplantation of purified astrocytes has been shown to promote axonal regeneration and functional recovery following acute transection injuries of the adult rat spinal cord (Davies et al., 2006). Similarly, other studies using iPSC-derived astrocytes have found that they can not only survive in the harsh post-injury environment but can produce improvements in the animals' sensory recovery (Hayashi et al., 2011). As a result, further work needs to be completed to determine if the spectrum of effects produce by astrocytes are a multimodal dose-response curve or the result of specific subpopulations of “good” and “bad” astrocytes.

### iPSC-derived perivascular stromal cells

Recently, our lab has shown that human fetal cadaveric perivascular stromal cells (pericytes) derived from the central nervous system (CNS-PSCs) have unique immunoregulatory functions that can reduce peripheral inflammatory cell infiltration and the permeability of the blood-spinal cord barrier after early intravenous injection (Badner et al., 2016). The disadvantage of CNS-PSCs is that they are not easily accessible and have a limited capacity to proliferate which limits their therapeutic efficacy. However, CNS-PSCs have been successfully generated from iPSCs and can potentially expand indefinitely without senescence (Quattrocelli et al., 2011; Orlova et al., 2014). Future studies can explore the potential of iPS-CNS-PSCs to improve behavioral outcomes after SCI.

## Challenges and recent progress for iPSC derived cells

In the decade since iPS technology was established, substantial progress has been made in developing safer and more efficient reprograming techniques, however, a few key challenges remain such as tumorigenicity and host immune rejection. Different induction methods can increase the rate of *de novo* mutations and chromosomal instability which raises the risk of tumorigenicity (Gore et al., 2011; Ji et al., 2012). It will be important to limit the level of mutagenesis to an acceptable limit prior to clinical use. Furthermore, undifferentiated iPSCs themselves or dedifferentiation of the cells pose a risk of teratoma formation. For this reason, efficient techniques to differentiate cells prior to transplantation, and remove potentially harmful cells after transplantation, will be important (Chen et al., 2013b). Several approaches are currently being developed for this purpose including *in vitro* removal of high proliferation rate cells, antibodies specifically targeting high-risk subpopulations (e.g., potentially teratoma-forming cells), and selective *in vivo* ablation of transplanted cells using suicide genes (Wu et al., 2014).

Immunogenicity is a concern with any cell therapy, however, the distinct advantages of iPSCs is their potential as an autologous source to reduce or eliminate immune rejection. In theory, autologous iPSCs derived from a non-invasive sampling of the patients' cells should avoid rejection by expressing markers of “self” with greater compatibility than any generally well-accepted allograft (e.g., group-matched blood). Importantly, this remains theoretical as human trials have yet to be conducted with autologous iPSCs. It will also be important to monitor changes in the expression pattern and level of the genes due to aberrant DNA methylation during reprograming process which could potentially evoke an immune responses in transplant recipients even when autologously derived (Zhao et al., 2011; Araki et al., 2013; Guha et al., 2013). If this is unavoidable, minor immunogenicity may be addressed using conventional immunosuppressives or short term monoclonal antibody-mediated co-stimulation/adhesion blockade of host T cells (Scheiner et al., 2014). As the technology develops, these risk and benefits, as well as new challenges, are likely to come to the forefront.

## Future directions

Stem cell transplantation for SCI is an attractive therapeutic strategy as the grafts offer multiple mechanisms to enhance recovery from this multifaceted disease process. Numerous preclinical studies have generated excitement around the use of iPSC-derived approaches with the added benefit of potentially generating autologous cells in the future (Wilcox et al., 2014). A limited number of clinical trials for the treatment of SCI using harvested stem cells have been performed in North America but none have yet used iPSC-derived cells. While the path to clinical translation has commenced, including the establishment of good manufacturing practices and animal-free media solutions, there are several major challenges that need to be further studied at the preclinical stage. These include the need to better understand the influence of epigenetics on iPSC-derived progeny and more refined techniques for differentiation/purification of cell lines. Furthermore, we require a deeper understanding of the graft-host microenvironmental interaction in SCI to tailor cell therapies to the recipient niche for maximal efficacy. Given the tremendous excitement around this technology, we foresee these challenges being overcome within the next several years to provide an effective regenerative therapy to patients with SCI.

## Author contributions

MK and CA: Literature review, manuscript writing, editing, and finalizing, approval of final manuscript. MF: Framing the concept and structure of the review, editing, and finalizing, approval of final manuscript.

## Funding

MF is supported by the Gerry and Tootsie Halbert Chair in Neural Repair and Regeneration and the DeZwirek Family Foundation.

### Conflict of interest statement

The authors declare that the research was conducted in the absence of any commercial or financial relationships that could be construed as a potential conflict of interest.
